# List of Reviewers 2023

**DOI:** 10.1002/ski2.383

**Published:** 2024-04-03

Successful and reliable publishing is dependent on expert, objective and timely peer review. The editorial team greatly appreciates the goodwill, time and effort that goes into writing these reviews and I would like to thank all those who reviewed for SHD last year.

Abbott, Rachel

Abdalla, Beatrice

Abdel Azim, Amira

Abdel Naser, Mohamed Badawy

Abdelmaksoud, Ayman

Abdo, Hamed

Aboobacker, Shamma

Abreu‐Velez, Ana

Abu El‐Hamd, Mohammed

Acar, Ayda

Agut‐Busquet, Eugenia

Ajani, Atinuke

Akasaka, Eijiro

Akhras, Victoria

Akiyama, Masashi

Albrecht, Joerg

Aldhalimi, Muhsin

Aleshin, Maria

Alhomida, Faris

Ali, Atif

Ali, Fatima

Allsop, Daniel

Almuqarrab, Fatimah

Alqurashi, Moayad M.

Altunay, Ilknur

Amalki, A.

Ansari, Farzana

Arents, Bernd

Argenziano, Giuseppe

Arif, Tasleem

Ashique, Karalikkattil

Atwan, Ausama

Azer, Professor Samy

Bagatin, Edileia

Bailey, Elizabeth

Bajwa, Dalvir

Balak, Deepak

Balieva, Flora

Barlow, Richard

Bassas‐Vila, Julio

Beattie, Paula

Beausillon, Chrstopher

Beniwal, Ranjana

Berg, Anna Korsgaard

Boch, Katharina

Borlu, Murat

Bose, Shiti

Brazzelli, Valeria

Brownell, Isaac

Bueno, Juan

Bulloch, Gabriella

Bunker, Christopher

Butt, Sanaa

Caldarola, Giacomo

Cartier, H.

Carvalho, Daniela

Cassarino, David S.

Chatproedprai, Susheera

Chauhan, Payal

Chernyshov, Pavel

Cho, Yung‐Tsu

Choi, Ellie

Choon, Siew Eng

Choudhary, Cherry

Cohen, Bernard

Conforti, Claudio

Cranwell, William

Criado, Paulo

Cruz, Adriano G.

Cuenca‐Barrales, Carlos

Curman, Philip

Daneshpazhooh, Maryam

Davies, Evelyn

Dawe, Robert

de Souza, Melanie Miyanji

Del Pozzo‐Magana, Blanca

Di Stefani, Alessandro

Diaz Calvillo, Pablo

Donetti, Elena

Dubash, Faisal

Dubois, Anna

Duffy, Jonathan

Dumic, Igor

Eberlein, B.

Elmasry, Maha Fathy

Elsaie, Mohamed

Elsayad, Khaled

Emtestam, Lennart

Fagan, Nicole

Faris, Ahmed

Farista, Arshi

Farrar, Mark

Feldman, Steven

Fernández Peñas, Pablo

Fujita, Yasuyuki

Fujiwara, Hiroshi

Furukawa, F

Furuta, Junichi

Fölster‐Holst, Regina

Gaglani, Bindi

Gallais Sérézal, Irène

Ganesh, G. Shankar

Gao, Xinghua

Gardner, Louis

Gawkrodger, David

Gershenwald, Jeff

Gilmore, Brendan

Godic, Aleksandar

Godor, Dorottya

González‐López, Marcos A.

Goodwin, Richard

Gopee, Nusayhah

Grantham, Henry

Griffiths, Tamara

Grijsen, Marlous

Gualdi, Giulio

Guest, Ella

Guo, Hao

Gupta, Aayush

Gutzmer, Ralf

Hampton, Philip

Haneke, Eckart

Harris, Charlotte

Haselgruber, Sofía

Hashimoto, Kenji

Hashizume, Hideo

Hay, Rod

Hayama, Koremasa

Heelan, Kara

Heerfordt, Ida M.

Hefez, Louise

Henderson, Stuart

Herbig, Michael

Herman, Anne

Hieta, Niina

Honnor, Kate

Hruza, George

Hsu, Chao‐Kai

Ibrahim, Shady

Inui, Shigeki

Ishii, Norito

Ishitsuka, Yosuke

Iwata, Hiroaki

Johnson, Oliver

Jackson, David

Jafferany, Mohammad

Janowska, Agata

Johansson, Peter

Johnson, Oliver

Jost, Marion

Kempf, Werner

Kennedy, Cornelis

Keurentjes, Anne

Khachemoune, Amor

Khunger, Niti

Kim, Hei Sung

King Stokes, Natalie

Kirby, Lisa

Kirtschig, Gudula

Kirwin, Thomas

Kishibe, Mari

Kluger, Nicolas

Knapp, Peter

Kose, Kivanc

Kosumi, Hideyuki

Kotb, Iman

Kravvas, Georgios

Kristensen, Johannes

Kumar, Piyush

Kurzhals, Jonas

Lada, Georgia

Langan, Ewan

Larocca, Cecilia

Lee, Peter

Lewis, Fiona

Li, S.C.

Lim, Chris

Lim, Su Ping Regina

Liu, Bo

Liu, Ze‐Hu

Lockwood, Katie

Long, Valencia

Lopes, Cristina

Luo, Di‐Qing

Lynch, Maeve

Mambwe, Bezaleel

Martins, Giselle

Matic, Aleksandra

Matsuda, Kazuki

Maza‐Ramos, Gibert

McCourt, Collette

McPherson, Tess

Megna, Matteo

Menezes, Antonio S., Jr.

Micallef, Daniel

Millette, Dijon

Miteva, Mariya

Miyagaki, Tomomitsu

Molina‐Leyva, Alejandro

Mondal, Himel

Montero‐Vilchez, Trinidad

Moss, Celia

Murphy, Ruth

Murray, Gregg

Muñoz‐Barba, Daniel

Naafs, Ben

Natsuga, Ken

Navarrete‐Dechent, Cristian

Negera, Edessa

Nelson, Amanda

Nico, Marcello

Nomura, Toshifumi

Novakovic, Ljuba

Nwabudike, Lawrence

Nylander, Elisabet

Oh, Choon Chiat

Ollague, Jose

Olsen, Catherine

Owen, Caroline

Palanivel, Janani A.

Paolino, Giovanni

Papanikolaou, Marieta

Papp, Kim

Parajuli, Niraj

Pasquali, Paola

Patel, Priya

Patra, Suman

Paudel, Vikash

Peng, Hsien‐Li Peter

Picardo, Mauro

Pirmohamed, Munir

Polo Silveira, Luísa

Powell, James

Pudasaini, Prajwal

Puri, Neerja

Rademaker, Marius

Ramien, Michele

Ramot, Yuval

Reich, Adam

Richmond, Jillian

Ridd, Matthew

Roesner, Lennart

Russo, Filomena

Saceda‐Corralo, David

Sachedina, Dilshad

Sadik, Christian

Salah, Eman

Salti, Giovanni

Sanchez‐Diaz, Manuel

Sarkari, Bahador

Schofield, Julia

Sebaratnam, Deshan

Selk, Amanda

Shah, Nasir

Shah, Reena

Sharma, saraswoti

Shimanovich, Iakov

Shinohara, Michi

Shiohara, Tetsuo

Shipman, William

Short, K.A.

Shukla, Prakriti

Sil, Abheek

Singh, Mini

Solimani, Farzan

Soto‐Moreno, Alberto

Sotto, Mirian

Soutou, Boutros

Starace, Michela

Steinberg, Julia

Sticherling, Michael

Svendsen, Mathias Tiedemann

Takahashi, Hayato

Takwale, Anita

Tan, Cher‐Han

Tatian, Artiene

Tejera‐Vaquerizo, Antonio

Temiz, Selami Aykut

Thangaraj, Prabha

Tokez, Selin

Tzellos, Thrasivoulos

Urena‐Paniego, Clara Amanda

Veierød, Marit B.

Veronese, Federica

Vilchez‐Marquez, Francisco

Vittrup, Ida

von Eije, Karin

Vorobyev, Artem

Walko, Gernot

Wan, Hong

Weatherhead, Sophie

Winn , Remus

Worsley, Peter

Xu, Shuai

Yazici, Serkan

Ye, Weiyu

Yell, Jennifer

Yélamos, Oriol

Zanini, Ludovica

Zasada, Malwina

